# Servant Leadership, Innovative Capacity and Performance in Third Sector Entities

**DOI:** 10.3389/fpsyg.2020.00290

**Published:** 2020-02-26

**Authors:** Felipe Hernández-Perlines, Luis Andrés Araya-Castillo

**Affiliations:** ^1^Department of Business Administration, University of Castilla-La Mancha, Toledo, Spain; ^2^Facultad de Economía y Negocios, Universidad Andrés Bello, Santiago de Chile, Chile

**Keywords:** servant leadership, innovative capacity, performance, Third Sector entities, structural equation modeling, fuzzy-set qualitative comparative analysis

## Abstract

This paper analyses the relationship between servant leadership, innovative capacity and performance in Third Sector entities and proposes a mediation model. This research is based on a two-fold theoretical approach: the servant leadership approach and the resource-based approach. The data have been obtained through a survey sent to territorial and functional managers of Third Sector entities. The fieldwork ran from June to September 2019. At the end of the entire process, 85 valid questionnaires were obtained. For the analysis of the results, a double methodology has been used: (1) a method of second order structural equations (PLS-SEM) and, (2) qualitative comparative analysis (QCA). The main contributions of this work are: 1) a double theoretical approach has been applied in this work, which has allowed to adequately define the relationships between servant leadership, innovation capacity and performance in Third Sector entities; (2) the application of a double data analysis methodology has allowed us to obtain robust and reliable results; (3) the measures of the three composites used (servant leadership, innovative capacity and performance) have adequate reliability and validity values; (4) the servant leadership positively influences the performance of Third Sector entities being able to explain the 35.6% of the variation of the performance of these entities and besides, it is a necessary condition for this performance to take place, (5) the average innovative capacity in the influence of the servant leadership in the performance of the entities of the Third Sector, being a necessary condition. Mediation is total, eliminating the direct effect of servant leadership on the performance of third Sector entities and increasing the capacity to explain the variation in the performance of Third Sector entities up to 44.7%.

## Introduction

The Third Sector is a fundamental part in the economic and social development model of many countries. Despite this importance, there are still few works that attempt to analyze the direction of this type of entity. With this work we try to analyze the performance in the third sector entities based on the servant leadership and the innovative capacity. To be able to keep playing this important role, Third Sector entities need to create action measures which allow them to overcome the challenges of a fast turning environment and, by doing so, secure their future viability and sustainability. This new framework means that Third Sector entities must implement new management models. One of these new models is the servant leadership approach. In recent years, servant leadership has become a management approach (Liden et al., 2008; van Dierendonck, 2011; Ruiz-Palomino et al., 2019) suitable for dealing with the changes that are taking place in an increasingly complex environment (Slåtten and Mehmetoglu, 2011). Third Sector entities are not unaware of these changes, so it is necessary to analyze whether this new approach can be used by Third Sector entities to improve their performance. Although the relationship between servant leadership and performance has been studied in other sectors (see Liden et al., 2008; Overstreet et al., 2014; Huang et al., 2016; Sousa and van Dierendonck, 2016; Hoch et al., 2018; Lee et al., 2019) it has not been addressed in Third Sector entities. In this sense, this work attempts to respond to the call from authors such as Ronquillo (2011) and Allen et al. (2018) to analyze servant leadership in Third Sector entities, raising the following research question: Does servant leadership influence the performance of Third Sector entities? Most of the previous works have measured performance from economic benefit or measures related to this benefit. The originality of this work is the way in which the performance of Third Sector entities has been measured, given that the objective of Third Sector entities is not economic benefit, so it is not easy to measure their performance with merely financial measures, and it is necessary to use other non-financial measures. Some authors suggest using a series of indicators to evaluate their activity, the degree of achievement of goals, the participation of other agents, etc. (AECA, 2012; Maguregui Urionabarrenechea et al., 2017). The originality of this work lies in measuring performance as a multidimensional variable in order to obtain integrated information on the management of this type of entities (Maguregui Urionabarrenechea, 2014).

The second aspect that we are going to analyze in this document is the innovative capacity. The innovative capacity is the ability to respond differently to the needs that companies, in general, and Third Sector entities, in particular. The innovative capacity measures the way in which new products, new processes and even new ideas are created (Hult et al., 2004). With this inclusion we try to respond to the call of Antonakis et al. (2010) and Eva et al. (2019) to include mediators in order to improve the understanding of the influence of service leadership on the performance of Third Sector entities. The consideration of the mediating effect of innovative capacity is due to the fact that it influences the servant leadership relationship (Waite, 2014) because it represents a different way of responding to the needs of Third Sector entities by creating new products, new processes and even new ideas (Hult et al., 2004) and involving both management (Katrinli et al., 2009) and employees (Chen and Huang, 2009). Based on the above, we pose the second research question: the average innovative capacity in the influence of employee leadership on the performance of Third Sector entities?

The theoretical approach behind this study is twofold. On the one hand, we use the servant leadership approach (Greenleaf, 1977; Graham, 1991; van Dierendonck, 2011; Liden et al., 2014; Eva et al., 2019) to analyze the behavior of managers toward employees and its effect on performance. On the other hand, we use a resource-based approach (Barney and Clark, 2007) to analyze how innovative capacity affects performance (Božic and Ozretic-Došen, 2015).

Data have been obtained through a survey sent to area and operation managers of Third Sector entities in Spain. The questionnaire contained general questions and questions on Servant Leadership, on Innovative capacity and on performance (see Appendix). The questions in the last three sections were asked using a Likert scale (1–7). The fieldwork took place from June to September 2019. The result of the whole process were 85 valid questionnaires.

This paper tries to answer the call made by Eva et al. (2019) to prove the validity of the correlation between servant leadership and performance by using different methods of data analysis. For this reason, in this study we use two methods to analyze the results: (1) partial least squares (PLS), which will enable us to assess the validity and reliability of the three variables in question, as well as their relationship and (2) Qualitative Comparative Analysis (QCA), which will enable us to determine the conditions under which servant leadership and innovative capacity translate into better performance in Third Sector entities. Ambos methods are suitable for this research because they will be used to analyze social phenomena using small data sets (Ragin, 2000, 2009; Henseler et al., 2009). Furthermore, the use of a double methodology for the data analysis will enable us to obtain more solid investigation findings (Hernández-Perlines et al., 2019a).

This work has contributed to the analysis of the performance of third sector entities on the basis of servant leadership and innovation capacity: if Third Sector entities want to improve their performance, they must take into account both servant leadership and innovative capacity.

## Theoretical Background and Research Hypotheses

### Servant Leadership

Servant leadership starts as the answer for businesses wishing to implement new leadership approaches and it focuses on employees’ social identity (Chen et al., 2015) and tries to gain their trust (Lee et al., 2019). servant leadership is a relatively new approach (Mittal and Dorfman, 2012). Research on servant leadership is divided into three stages (Eva et al., 2019): (1) the first stage – which we will call conceptual development – with projects that try to provide an initial idea of servant leadership. In this stage, the works of Greenleaf (1977) and Spears (1996) are particularly noteworthy. (2) The second stage, which we will call measurement, with projects that attempt to establish a measurement of servant leadership. In this stage, the works of Lytle et al. (1998), Page and Wong (2000), Ehrhart (2004) amongst others, are particularly noteworthy. (3) The third stage, which we will call model development, where we can find many works which represent sophisticated models which can be used to analyze the background of servant leadership, its relationship with performance and even the analysis of mediation and/or moderation factors. In this stage, the works of Liden et al. (2014), Flynn et al. (2016), Sousa and van Dierendonck (2016), Sendjaya et al. (2019) are particularly noteworthy.

Literature on servant leadership has focused on achieving a unanimous definition and on how to measure it (Grisaffe et al., 2016; Newman et al., 2018; Lee et al., 2019). We agree with Eva et al. (2019) that the majority of definitions of servant leadership only offers partial descriptions of servant leaders’ behavior (what, why and how they behave with employees). We can therefore state that there isn’t a generally accepted definition (van Dierendonck, 2011; Parris and Peachey, 2013), which results in significant conceptual confusion (Winston and Fields, 2015). Thus, servant leadership begins with the leader’s wish to serve and, by doing so, ease the employees’ Performance (Greenleaf, 1977). Servant leadership prioritizes the needs of employees (Liden et al., 2014) and turns them into its goal (van Dierendonck, 2011; Eva et al., 2019). Servant leadership focuses on the employees’ well-being (van Dierendonck, 2011; Liden et al., 2014; Winston and Fields, 2015) trying to effectively satisfy their needs (Chiniara and Bentein, 2016), their development and their empowerment (van Dierendonck, 2011), even above the interests of the actual leaders (Hale and Fields, 2007). For this reason, servant leadership is a top-down approach (Duren, 2017) which involves multiple stakeholders (Lemoine et al., 2019) showing concern toward others (Eva et al., 2019), both within and out of the organization (Zohar, 2005; Liden et al., 2008). It is a suitable approach for the analysis of the behavior of Third Sector entities’ managers (Herman, 2016), as it focuses on the qualities and behavior of said managers (Ronquillo, 2011; Herman, 2016). Moreover, servant leadership in Third Sector entities empowers its servants by providing them with the necessary tools, developing their skills and encouraging them to take part in decision-making processes (Ebener and O’Connell, 2010).

The organizational performance measures how and when the firm meets its previously specified objectives. In this respect, the leadership is an important factor for the company to be able to improve on its results and (Karamat, 2013) and achieve a sustainable competitive advantage (Rowe, 2001). Therefore, servant leadership is positively related to performance and said relationship is maintained even when using a multidimensional performance measurement (Liden et al., 2008; Overstreet et al., 2014; Huang et al., 2016; Sousa and van Dierendonck, 2016; Hoch et al., 2018; Lee et al., 2019). Based on the aforesaid, we can formulate the following hypothesis (proposition):

H_1_ (Proposition _1_): Servant leadership has a positive effect on Third Sector entities’ performance.

### Innovative Capacity

Innovative capacity can be defined as the ability of the firm to create and develop new products, new processes or new ideas with which to achieve a competitive advantage in a constantly changing environment (Hult et al., 2004; Alegre et al., 2005; Helfat and Martin, 2015; Feng et al., 2018), which can be crucial for its survival. Based on their innovative capacity, firms achieve a competitive advantage because they can detect in advance new opportunities in their surroundings (Gunday et al., 2011; Yap et al., 2018). Innovative capacity means a proactive behavior in order to take advantage of the opportunities that arise in a turbulent environment (Droge et al., 2008). Innovative firms or organizations are known for encouraging a creative behavior and an exchange of information amongst their employees (Wong and Tong, 2012), who are the source of the company’s innovation (Yuan and Woodman, 2010).

The mediating role of innovative capacity has already been described by authors such as Obiwuru et al. (2011) and Eva et al. (2019) amongst others, when they state that the relationship between leadership and performance requires the firm to be innovation oriented. Furthermore, competitive advantage is determined by the fact that leaders promote creativity amongst their employees (Neubert et al., 2008). Based on the above, we formulate the following mediation hypothesis (proposition):

H_2_ (Proposition_2_): The innovative capacity mediates the relationship between servant leadership and performance of the entities of the Third Sector.

The analysis of the mediation effect of innovative capacity of servant leadership in the performance of Third Sector entities implies a double consideration, following the recommendations of Cepeda-Carrión et al. (2017): 1) on the one hand, an analysis of the of servant leadership in innovative capacity and, 2) on the other hand, an analysis of the of innovative capacity in performance.

### Analysis of the of Servant Leadership in Innovative Capacity

We define innovative capacity as the tendency to encourage new ideas (Katrinli et al., 2009) that can then be put into practice (Lumpkin and Dess, 1996) considering the cultural facet of it (Hult et al., 2004), which allows the leader to act (Dackert et al., 2004). In this sense, the relationship between servant leadership and performance is determined by the innovative capacity, which in turn is influenced by the employees’ ability to generate new ideas, new work systems, new services, etc. (Yuan and Woodman, 2010; Goepel et al., 2012). As a result, the servant leader can encourage innovative behavior amongst employees (Liden et al., 2008; Panaccio et al., 2015; Williams et al., 2017; Cai et al., 2018) so as to achieve a positive impact on innovation (García-Morales et al., 2008; Gumusluoglu and Ilsev, 2009; Panaccio et al., 2015) and on innovative capacity (Neubert et al., 2008; Schaubroeck et al., 2011; Yoshida et al., 2014). Previous researches show that companies led by servant leaders tend to improve their innovative capacity (Yoshida et al., 2014; Ruiz-Palomino et al., 2019). Based on the above, we formulate the following hypothesis model:

H_2__a_ (Proposition_2__a_): Servant leadership has a positive effect on innovative capacity in Third Sector entities.

### Analysis of the Impact of Innovative Capacity on Performance

In order to achieve the objectives of any organization, also in third sector entities, it is necessary to consider the different interest groups. In this sense, management processes tend to be very similar in all types of organizations, although the characteristics of each type of organization must be taken into account. In this work we analyze the innovative capacity as it is a fundamental determinant of competitive advantage (Schumpeter and Opie, 1934) and a cultural element that managers can control to a great extent (Hult et al., 2004). In addition, innovative capacity remains a topic of academic and organizational interest (Hughes et al., 2018). The capacity to innovate makes it possible to improve a company’s competitiveness in an environment that is subject to major changes (Hult et al., 2004; Alegre et al., 2005; Helfat and Martin, 2015; Feng et al., 2018), and is even crucial for its survival (Hernández-Perlines et al., 2019b). The capacity for innovation is a behavioral variable linked to the company’s willingness to change (Calantone et al., 2002), an organizational attitude necessary to be open to new ideas (Crespell and Hansen, 2008). This need for change is common to both market firm and Third Sector entities. However, the social responsibility of this type of entity, strategic and financial restrictions determine innovative behavior in Third Sector entities (Hull and Lio, 2006). Innovation oriented organizations tend to favor creativity (Neubert et al., 2008) by attaching greater value to innovation (Yuan and Woodman, 2010). In the case of Third Sector organizations, the most relevant innovation is usually process innovation (Hull and Lio, 2006). The innovative capacity tends to produce an improvement in the performance of any type of organization (Damanpour and Gopalakrishnan, 2001; Rosenbusch et al., 2011; Ruiz-Palomino et al., 2019), which is why we can state the following hypothesis.

H_2__b_ (Proposition_2__b_): Innovative capacity has a positive impact on the performance of Third Sector entities.

**FIGURE 1:**
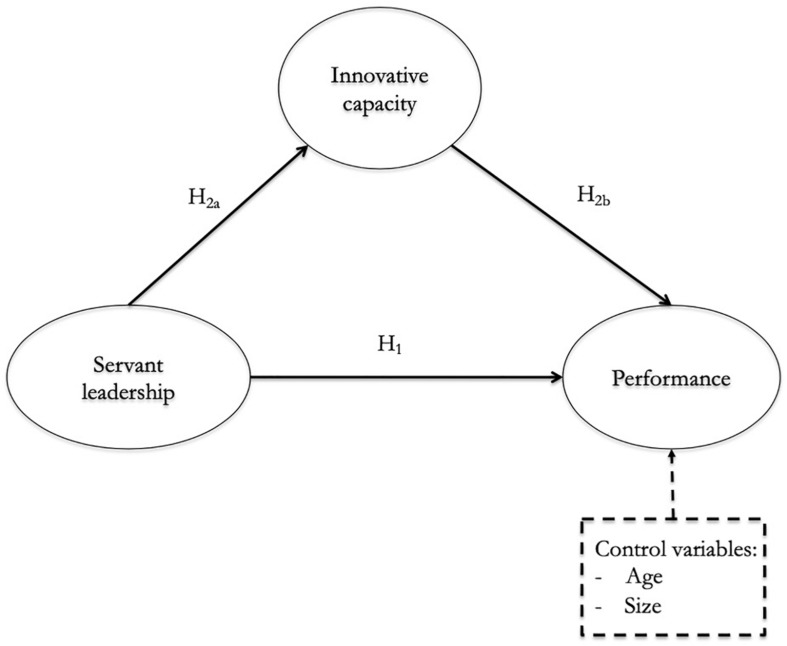
Investigation model.

Once the different hypotheses have been formulated, the research model would be the one shown in Figure 1.

## Materials and Methods

### Sample

The data was obtained through a survey sent by e-mail to the directors of Third Sector entities in Spain. After a pilot test of the questionnaire with a group of managers from these two organizations and academic experts, the clarity, comprehension, legibility and adequacy of the questions asked were confirmed. When designing the questionnaire, the advice of Podsakoff et al. (2003, 2012) was taken into consideration so as to mitigate both social convenience bias (the e-mail was sent together with an introduction letter explaining the research aim, ensuring the confidentiality and anonymity of the respondent) as well as the common method bias (simple, specific, and unambiguous questions). The fieldwork took place between June and September 2019 and, at the end of the whole process, we obtained 85 valid questionnaires.

The paper study collected data on both the exogenous and endogenous variables from the same respondents at one point in time and using the same instrument, thus potential common method variance as false internal consistency might be present in the data. We tested for common methods bias to establish that such bias did not distort the data we collected. To do so, we used two approaches. First, we examined the exploratory, unrotated factor analysis to find the results of Harman’s single factor test for all of the first-order constructs using a standard statistical package. The aim of the test is to determine whether a single factor emerges that explains the majority of the variance in the model. If so, then common method bias likely exists on a significant level (Lowry and Gaskin, 2014). The result of our factor analysis produced 14 distinct factors, the largest of which accounted for 42.35% variance explained by a single factor. This shows that the common method bias was not a major concern in this study (less than 50% cut-off point). In a second approach, we analyzed the common latent factor (CLF) to capture the common variance among all observed variables in the model. Adding a first-order factor to all observed items in the model and comparing the standardized regression weights from this CFA model to the standardized regression weights of a model without the CLF (Gaskin, 2017), the results show that all the values are similar (the difference is less than 0.2). As such, common method bias was not a major threat in our data set.

The most relevant data of the fieldwork can be found in Table 1.

**TABLE 1:** Details of the sample and data collection.

Property	Value
Context	Spain
Responses received	85
Sampling method	Simple random
Confidence level	95%, *p* = *p* = 50%; α = 0.05
Statistical Power	0.888
Data collection period	June to September 2019

To overcome representativeness problems in the sample, its statistical power was measure with Cohen’s retrospective test (1992), using the stats program G ^∗^ Power 3.1.9.2 (Faul et al., 2009). In our case, the sample’s statistical power was 0.888, higher than the 0.80 limit set by Cohen (1992).

### Measurement of Variables

For servant leadership, innovative capacity and performance, a 7-point Likert scale response format (1 – strongly disagree, 7 – completely agree) was used.

#### Servant Leadership

Servant leadership was measured using a ten-item scale proposed by Winston and Fields (2015). This variable was made operative as a first-order composites type a.

#### Innovative Capacity

Innovative capacity was measured using three items as per Keh et al. (2007). innovative capacity was made operative as a first-order composites type a.

#### Performance

Performance was measured using the scale proposed by Maguregui Urionabarrenechea et al. (2017). Performance was made operative as a second order composites type a. Performance, as a multidimensional measure, was defined on the basis of four Performance categories that are tailored to the characteristics of Third Sector entities. Specifically, in this work, Performance was analyzed from four perspectives: (1) from the users/beneficiaries; (2) from social activity; (3) from training and development and, (4) from financial resources.

#### Control Variables

As control variable we have used Size, that is the number of employees and age, that is the number of years that the organization has been in operation.

### Data Analysis

To prove these hypotheses (propositions) and analyze both the direct effect and the mediating effect, this research has used a double methodology:

(a) On the one hand, a partial least square method (PLS). As a multivariate quantitative method of structural equations, PLS allows research questions to be addressed for three reasons: (1) it has a predictive nature (Sarstedt et al., 2014; Hair et al., 2017), (2) it enables the observation of different casual relations (Jöreskog and Wold, 1982; Astrachan et al., 2014) and, (3) it is less demanding with the minimum size of the sample (Henseler et al., 2015). The software used for data analysis through SEM-PLS was SmartPLS v.3.2.8 (Ringle et al., 2015).
(b) On the other hand, a qualitative comparative analysis of fuzzy sets (fsQCA) was carried out because of their ability to adequately handle uncertainty and because they are suitable for studying social phenomena using small samples (Ragin, 2000, 2008). In addition, from the literature review we found an increasing number of papers using this data analysis technique (Fiss, 2007; Kraus et al., 2017; Huarng et al., 2020). This analysis enables us to: (1) overcome the problems associated with the minimum size of the sample (Rihoux and Ragin, 2004; Rihoux, 2006), (2) identify the effect of the combinations -configurations- of necessary and sufficient explanatory characteristics which other approaches are not able to adequately identify (Schneider and Wagemann, 2012), (3) offer a qualitative model based on set theory and logic (Thygeson et al., 2012), which complements quantitative techniques by modeling complex asymmetric relationships (Woodside, 2013) and, (4) allows to solve different types of problems (Huarng et al., 2020) as it allows to explain a result from the necessary or sufficient conditions (Ragin, 2000; Huarng et al., 2020). The software used for the comparative qualitative analysis of fuzzy sets (fsQCA) was fsQCA v. 3 (Ragin and Davey, 2016).

The research results will be presented in accordance with the methods of analysis that has been used. Therefore, first of all, the results will be analyzed according to the second-generation structural method (PLS-SEM) and subsequently the findings obtained from a quantitative comparative analysis methods (fsQCA) can then be exhibited.

### PLS-SEM Results

First, an analysis of the results and a comparison of hypotheses through a second-generation structural equation method (PLS-SEM) were carried out using the SmartPLS v.3.2.8 software (Ringle et al., 2015). First of all, it is important to ensure that all used variables are reliable and have adequate levels of convergent and discriminant validity. In order to achieve this Barclay et al. (1995); Roldán and Sánchez-Franco (2012), and Hair et al. (2017) suggest evaluating the measurement models using the following indicators:

(1) Composite reliability – Fornell and Larcker (1981) recommend values above 0.7 for composite reliability. According to Hair et al. (2019) these values are deemed “good” because they are between 0.7 and 0.9. Furthermore, they do not pose any redundancy problems as they never go above 0.95 (Drolet and Morrison, 2001; Diamantopoulos et al., 2012). The different variables have suitable composite reliability values (see Table 2).
(2) Cronbach’s alpha – Fornell and Larcker (1981) recommend Cronbach’s alpha values higher than 0.7. As shown in Table 2, Cronbach’s alpha exceeds these thresholds.
(3) Rho A – is used to calculate a reliability value that lies between the two previous extreme values (composite reliability and Cronbach’s alpha). Rho A, proposed by Dijkstra and Henseler, 2015, should be above 0.7 (Dijkstra and Henseler, 2015) and should be between the reliability values of the composites and Cronbach’s alpha (Hair et al., 2019). In our case this is met (see Table 2).
(4) AVE (Average Variance Extracted) – The AVE enables us to evaluate the convergent validity of the different components. Fornell and Larcker (1981) recommend a value above 0.5 for the AVE. In our case this is met (see Table 2).
(5) We can also analyze the discriminant validity by verifying that the correlations between each pair of constructs do not exceed the value of the square root of the AVE of each construct and the HTMT index. For discriminant validity to be met, the values of the HTMT ratio must be lower than 0.85 (Henseler et al., 2015). As shown in Table 2, there is discriminant validity, as the two previous aspects are fulfilled.

**TABLE 2:** Correlation matrix, composite reliability, convergent and discriminant validity, heterotrait-monotrait ratio (HTMT), and descriptive statistics.

Composite/Measure	AVE	Composite reliability	SL	IC	P
1. Servant leadership	0.735	0.874	0.857*		
2. Innovative capacity	0.697	0.863	0.639	0.834*	
3. Performance	0.701	0.857	0.571	0.652	0.837*
**Heterotrait-monotrait ratio (HTMT)**					
1. Servant leadership			0.564		
2. Innovative capacity			0.571	0.588	
3. Performance			0.601	0.583	0.571
Cronbach’s Alpha			0.849	0.762	0.741
Rho A			0.857	0.802	0.797
Media			4.34	4.06	4.18
Standard deviation			0.97	1.05	0.92

The correlations are for the second-order CFA output. *The elements on the diagonal show the square root of the AVE. AVE, average variance extracted; SL, servant leadership; CI, innovative capacity; P, performance.

Once the convergent and discriminant validity of the measurement model is ascertained, we proceed to test the hypotheses raised in the model. To do this, we must check the values of the trajectory coefficient and the level of significance, applying a bootstrapping procedure of 5,000 subsamples. To be able to determine the different effects, we follow the steps suggested by Hair et al. (2014) in order to apply Preacher and Hayes’s (2008) in the proposed mediation model.

**FIGURE 2:**
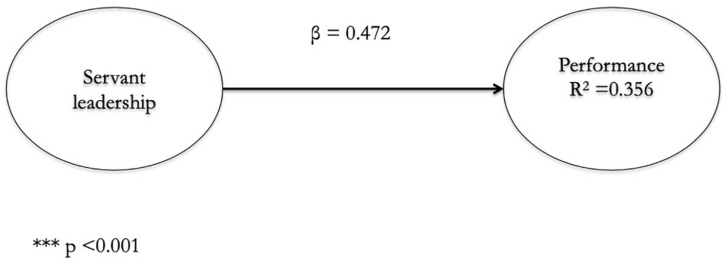
Direct model between servant leadership and the performance of Third Sector entities.

Firstly, we analyze the direct effect of servant leadership on the performance of Third Sector entities. Based on the bootstrapping procedure of 5000 subsamples, we calculate the values of the trajectory of the coefficient and its meaning. As we see, the effect is positive and significant (β = 0.472; *p* < 0.001) (see Figure 2 and Table 3). This result is reinforced by applying the percentile method to the bootstrap resampling with a 95% confidence rate (see Table 3). In addition, servant leadership is able to explain 35.6 percent of the variation in the performance of Third Sector entities. Therefore, the first of the hypotheses that stated that servant leadership has a positive influence on the performance of Third Sector entities is confirmed.

**TABLE 3:** Structural model.

	Path Coefficient (b)	*t*-value (bootstrap)	Confidence intervals 95%		Support
			Infer.	Super.	
**Direct model SRMR: 0.074**					
Total effect of SL on P (*c)*	0.472***	3.871	0.279	0.534	Yes
**Mediation model SRMR: 0.063**					
SL → P = *c′* (direct effect of LS on P)	0.072^ns^	0.692	0.010	0.361	No
SL → IC → P = *a*_1_*b*_1_ (vía IC) (total indirect effect of LS on P, mediation)	0.253		0.183	0.486	
H_2_ = SL → IC = *a*_1_	0.541***	5.439	0.458	0.721	Yes
H_3_ = IC → P = *b*_1_	0.468***	4.732	0.349	0.593	Yes

***p < 0.001, based on *t* (4,999), one-tailed test; ns, not significant. SL, servant leadership; IC, innovative capacity; P, performance.

**FIGURE 3:**
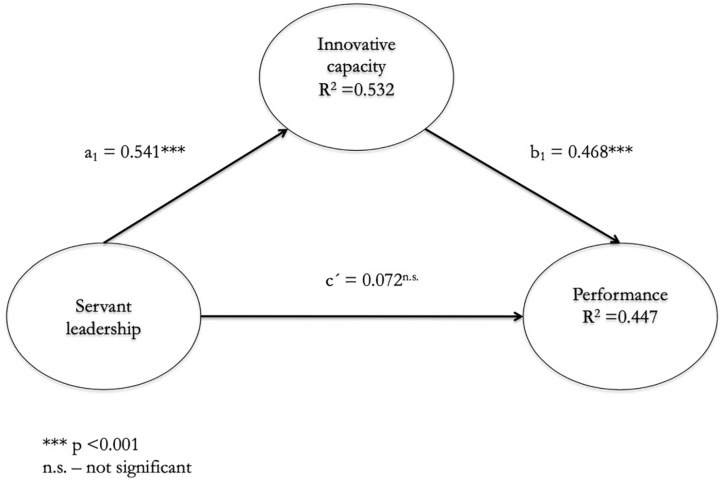
Model for mediating the innovative capacity in the relationship between servant leadership and the performance of Third Sector entities.

The second step is to include the effect of innovative capacity as a mediating variable. To do this, we first test the influence of servant leadership on innovative capacity. We observe that this effect is positive and significant (β = 0.541; *p* < 0.001) (see Figure 3 and Table 3). Secondly, we tested the influence of the innovative capacity and the performance of Third Sector entities. Again, this influence is positive and significant (β = 0.468; *p* < 0.001) (see Figure 3 and Table 3). Consequently, hypotheses H_2__*a*_ and H_2__*b*_ are confirmed. Furthermore, the mediating effect completely suppresses the direct effect of the servant leadership and the performance of the Third Sector entities, since the path coefficient is 0.072 and is not significant. On the other hand, the indirect effect of the Servant Leadership on the performance of the entities of the Third Sector through the innovative capacity is 0.253 (a_1_b_1_).

In this model, servant leadership is able to explain 53.2% of the variance of innovative capacity and this, in turn, is able to explain 44.7% of the variance of the performance of Third Sector entities. Therefore, there is a mediation of innovative capacity in relation to the servant leadership and the performance of Third Sector entities and said effect is total as it suppresses the direct effect (Baron and Kenny, 1986; Cepeda-Carrión et al., 2016).

Control variables, size and age, enable us to analyze the common variance among predictors and avoid overestimated parameters. However, a comparison of the results of three different statistical analyses – one that includes all control variables, another that includes only the control variables that are significantly related to our dependent variable and a third one without any control variables- revealed almost identical parameters, without any changes in the significance levels and confidence intervals. On the basis of the above and following Bernerth and Aguinis’s (2016), no control variable was included in our analysis.

By comparing the two models and bearing in mind the quality parameters, we can state that the mediation model is better than the direct model: the SRMR (Standardized Root Mean Square Residual). The model obtains a SRMR of 0.074 and the mediation model a SRMR of 0.063, in both cases below the threshold established by Henseler et al. (2015). This would imply additional support for the mediating role of absorptive capacity.

### fsQCA Results

In this section the results of the propositions based on a quantitative comparative method (fsQCA) will be analyzed using the software fsQCA 2.5 (Ragin and Sean, 2014).

In order to proceed with the fsQCA analysis, the Likert (1–7) responses were transformed into fuzzy-set responses. First of all, all lost cases must be eliminated (there were no lost cases in our sample). Then, all components must be calculated by multiplying the scores of the items they are made of. Subsequently, answers need to be recalibrated taking the three thresholds into consideration (Woodside, 2013): 10th percentile (under agreement or totally outside the category), 50th percentile (intermediate level of agreement or neither inside nor outside the category) and 90th percentile (high agreement or entirely within the category). Finally, necessity and sufficiency analyses were carried out to evaluate the effect of the different components. This sequence has been used in previous works such as Ruiz-Palomino et al’s. (2019). The fsQCA models generate three possible solutions: complex, parsimonious and intermediate. Following Ragin’s (2008) the intermediate solution was used in this work.

Based on the results that have been obtained (see Table 4), both servant leadership and innovative capacity, each on their own merit, are essential conditions for a performance improvement as they have a consistency value higher than a 0.90 which is the threshold established by Ragin (2008). Although servant leadership has a higher consistency value (0.947628) than innovative capacity (0.921567), it is also capable of explaining a higher performance percentage in Third Sector entities (83.78% of the performance). This enables us to state that the performance of Third Sector entities can be explained, to a greater degree, by servant leadership (in 83.78% of cases) than by innovative capacity (in 73.98% of cases). On the other hand, if we consider servant leadership and innovative capacity together, the consistency increases to 0.96 and it is able to explain 90.68% of the performance of Third Sector entities.

**TABLE 4:** Necessary conditions: intermediate solution.

	Outcome: Performance	
	Consistency	Coverage
SL	0.947628	0.837832
IC	0.921567	0.739824
SL * IC	0.968245	0.906828

*SL, servant leadership; IC, innovative capacity; P, performance.*

As per Eng and Woodside (2012), when consistency is higher than 0.75, the model in fsQCA is informative. In our case, the sufficiency condition demonstrates that the performance of Third Sector entities takes place when we consider servant leadership and innovative capacity (see Table 5). In other words, innovative capacity positively mediates the effect of servant leadership in the performance of Third Sector entities, as it is able to explain 90.98% of performance cases of Third Sector entities.

**TABLE 5:** Intermediate solution for the analysis of sufficient conditions.

	Outcome: Performance		
	Raw coverage	Unique coverage	Consistency
SL	0.845688	0.845688	0.746721
IC	0.832841	0.832841	0.728659
SL * IC	0.897650	0.897650	0.909864

*SL, servant leadership; IC, innovative capacity; P, performance.*

## Discussion

The study of Third Sector entities is increasingly important due to the relevance that these entities have in the economic and social development of many countries. Spain is no exception. For these entities to continue to play this important role, it is necessary for them to implement new management models that allow them to adapt to the changing conditions of the environment. In this sense, both servant leadership and the innovative capacity may be aspects that Third Sector entities should take into account in order to improve their performance levels. Servant leadership has become a new approach for facing up to the changes that are taking place in the environment and in the organizations themselves (Slåtten and Mehmetoglu, 2011). The behavior of Third Sector entities can also be analyzed on the basis of servant leadership (Herman, 2016). In this sense, in this work we try to verify the influence of servant leadership on the performance of third sector entities. The way to measure performance in Third Sector entities is not the same as in for-profit organizations. The purpose of Third Sector entities is not to maximize economic benefit, but rather to achieve non-economic objectives linked to their purpose (Ortiz-Gómez et al., 2020). In this sense, we consider performance as a multidimensional measure that takes into account budgetary, social, training and user aspects (Maguregui Urionabarrenechea et al., 2017). One of the objectives of this research is to analyze the influence of servant leadership on the innovative capacity of Third Sector entities. With this objective in mind, we try to respond to the call made by Ronquillo (2011) and Allen et al. (2018) to continue studying server leadership in Third Sector organizations in greater depth. Most of the previous studies focus on the influence of servant leadership on performance as a quantitative variable (Liden et al., 2008; Overstreet et al., 2014; Huang et al., 2016; Sousa and van Dierendonck, 2016; Hoch et al., 2018; Lee et al., 2019). The originality of this work is that performance has been considered as a subjective multidimensional variable, which is obtained from the assessment on a Likert scale (1–7) of 85 managers of Third Sector entities.

The relationship between servant leadership and the performance of Third Sector entities is quite complex, which is why authors such as Antonakis et al. (2010) and Eva et al. (2019) call for a more profound analysis, including mediators. These mediators will make it possible to improve the understanding of the aforementioned relationship. In our study we opted for the innovative capacity. This inclusion is motivated by the fact that the environment is subject to great changes, to which Third Sector entities are not alien. To better adapt to these changes, many organizations use innovation to respond to these changes in a different way (Hult et al., 2004). This process must involve both management (Katrinli et al., 2009) and workers (Chen and Huang, 2009). Although product innovation is not usually very common in Third Sector entities, we do find a greater relevance of process innovation or new ideas in these entities (Hull and Lio, 2006), above all due to the direct relationship with users. Creativity is a relevant factor in this type of entity to satisfy new user needs.

To test the research questions formulated, we use two research methods: on the one hand, a method of second generation partial least squares structural equations (PLS-SEM) and, on the other hand, a method of qualitative comparative analysis (fsQCA). With the use of this double methodology we try to respond to the call of Eva et al. (2019) to use different methods of analysis for the validity of the servant leadership relationship in the performance of third sector entities.

This study gives an affirmative answer to the first research question, that is, that servant leadership positively influences the performance of Third Sector entities. In this sense, the results obtained coincide with previous studies that use quantitative measures of performance (Liden et al., 2008; Overstreet et al., 2014; Huang et al., 2016; Sousa and van Dierendonck, 2016; Hoch et al., 2018; Lee et al., 2019). The most relevant contribution of this work is the positive influence of servant leadership on performance, regardless of how we measure that performance. This work allows us to affirm that servant leadership positively influences the performance of Third Sector entities, even if we use a subjective measure of such performance.

This research allows us to say that mediation role of the innovative capacity in the effect of the servant leadership in the performance of the entities of the Third Sector. The analysis of the measurement of the innovative capacity must be carried out in two phases or stages. In the first place, we must check the influence of the servant leadership on the innovative capacity. This work allows us to state that servant leadership has a positive influence on the innovative capacity of Third Sector entities, coinciding with other research (Neubert et al., 2008; Schaubroeck et al., 2011; Yoshida et al., 2014). Secondly, we must analyze the influence of innovative capacity on performance. The results of this research allow us to state that the innovative capacity has a positive influence on the performance of Third Sector entities. Therefore, if the entities of the Third Sector want to improve their performance, their managers must be aware of exercising a leadership centered on the workers, that encourages the generation of new ideas, creativity, etc. In short, they have to act on the innovative behavior of their workers to promote the innovative capacity and with it, to improve their levels of performance.

## Conclusion

With this work we have deepened the analysis of the servant leadership in the Third Sector entities. The main conclusion of this work is that Third Sector entities can use the servant leadership approach to improve their performance, allowing them to analyze their behavior (Herman, 2016). Therefore, servant leadership is an antecedent of the performance of third sector entities, positively influencing it. The application of servant leadership will allow Third Sector entities to align the objectives of the Third Sector entity with those of its employees and the end users of its activity. With this result, we give an affirmative answer to the first of the research questions and fulfill one of the objectives of this work of analyzing servant leadership, responding to the call by Ronquillo (2011) and Allen et al. (2018).

The relationship between servant leadership and the performance of Third Sector entities is not easy to analyze, due to the very characteristics of this type of entity and because its purpose goes beyond the achievement of economic objectives. In order to collect the objectives of different stakeholders that act in third sector institutions, we have considered a multidimensional measure of performance. Given the complexity of the relationship between servant leadership and performance in Third Sector entities, it is necessary to consider other factors that may influence this relationship. In our case, given that it is necessary for third sector organizations to adapt to the changes that are taking place in their environment, we have considered analyzing the effect of innovative capacity on the relationship between servant leadership and performance. Specifically, the mediating effect of innovative capacity is analyzed in order to respond to the call from authors such as Antonakis et al. (2010) and Eva et al. (2019) to include mediators in this analysis. The results obtained in this work allow us to assert that the average innovation capacity in the influence of servant leadership on the performance of Third Sector entities. With this conclusion we respond to the second objective of this work. Furthermore, mediation is total and when innovative capacity is considered, the direct relationship of servant leadership in the performance of Third Sector entities is annulled. In short, for servant leadership to influence the performance of Third Sector entities through the innovative capacity. The above implies that servant leadership influences the innovative capacity and the latter influences the performance of Third Sector entities.

The main implication of this work for the management of Third Sector entities is that in order to improve their performance they must apply new management systems that consider both servant leadership and innovative capacity.

## Limitations and Future Research Lines

This work has the following limitations, the solution of which can lead to future research works. The first limitation is related to the number of questionnaires obtained. Although the research was carried out with sufficient statistical strength, *y* data analysis methods have been used to achieve significance with few observations and to draw generalizable conclusions (Fedriani-Martel and Romano-Paguillo, 2017; Hair et al., 2019), an increase in the number of managers surveyed would be useful.

The second limitation comes from the fact that neither the employees (servants) nor the beneficiaries of Third Sector entities were taken into consideration. In future researches the field of study could be extended to other collectives or interest groups linked to Third Sector entities.

As future lines of research *per se*, we can point out studies which compare these relationships with other type of Third Sector entities such as corporative foundations, religious organizations, social associations, NGOs, social cooperatives, etc.

Another future investigation line would be comparing the results of Third Sector entities with those of firms within the free market economy.

Finally, it would be interesting to carry out longitudinal studies to observe how the relationships obtained between the variable in question evolve over time and check the causal relationships between themselves.

## Data Availability Statement

The raw data supporting the conclusions of this article will be made available by the authors, without undue reservation, to any qualified researcher.

## Ethics Statement

Ethical review and approval were not required for the study on human participants in accordance with the local legislation and institutional requirements. The patients/participants provided their written informed consent to participate in this study.

## Author Contributions

All authors listed have made a substantial, direct and intellectual contribution to the work, and approved it for publication.

## Conflict of Interest

The authors declare that the research was conducted in the absence of any commercial or financial relationships that could be construed as a potential conflict of interest.

## Appendix

### Server Leadership Questionnaire

**Table:**

How many years you’ve been a director?	Age of the respondent:
Sex of respondent: Male Female	Respondent’s level of education:
Year of appointment:	Number of employees under your direction:
He’s worked at another entity before?	What sector?
He has worked before in another position at the entity where he currently works: Yes No	

### Instructions

Evaluate the importance of the following factors. Mark with an X the assessment you consider appropriate. If you do not agree at all, mark 1, and when you feel you agree completely, mark 7.

### Server Leadership

**Table:**

Practice what you preach	①	②	③	④	⑤	⑥	⑦
It serves people regardless of their nationality, gender or race, etc.	①	②	③	④	⑤	⑥	⑦
Sees serving as a mission of responsibility to others	①	②	③	④	⑤	⑥	⑦
He’s genuinely interested in the employees as people	①	②	③	④	⑤	⑥	⑦
Understand that serving others is the most important thing.	①	②	③	④	⑤	⑥	⑦
He’s willing to sacrifice himself to help others	①	②	③	④	⑤	⑥	⑦
It seeks to instill confidence rather than fear or insecurity	①	②	③	④	⑤	⑥	⑦
It’s always honest	①	②	③	④	⑤	⑥	⑦
It aims to contribute more to society with its work	①	②	③	④	⑤	⑥	⑦
It promotes values that transcend self-interest and material success	①	②	③	④	⑤	⑥	⑦

### Innovative Capability

**Table:**

It considers that the degree of novelty of the latest products and services is high	①	②	③	④	⑤	⑥	⑦
It considers that the latest technological innovations are used in new products and services	①	②	③	④	⑤	⑥	⑦
It considers that the speed of development of new products and services is high	①	②	③	④	⑤	⑥	⑦
It considers that a very high number of new products and services have been introduced	①	②	③	④	⑤	⑥	⑦
It considers that his entity has been the first to introduce a very high number of new products and services	①	②	③	④	⑤	⑥	⑦
It considers that the technological competitiveness of its very high	①	②	③	④	⑤	⑥	⑦
It considers that the speed with which they adopt the latest technological innovations in their processes is very high	①	②	③	④	⑤	⑥	⑦
It considers that the degree of novelty of the technologies used in the processes in his entity is very high	①	②	③	④	⑤	⑥	⑦
It considers that a very high number of new processes or techniques have been introduced/started in my entity	①	②	③	④	⑤	⑥	⑦

### Performance Indicators

#### User/Beneficiary Perspective

**Table:**

To know and improve the beneficiaries’ perception of the quality of the service provided	①	②	③	④	⑤	⑥	⑦
Improve coverage of the needs of the beneficiaries served	①	②	③	④	⑤	⑥	⑦
Improve coverage of social needs in the area	①	②	③	④	⑤	⑥	⑦
To promote the image and increase the presence of the entity among the beneficiary users	①	②	③	④	⑤	⑥	⑦
To promote the image and increase the presence of the entity among the beneficiary users	①	②	③	④	⑤	⑥	⑦

### Financial Resource Perspective

**Table:**

To detect possible deviations in the budgetary application	①	②	③	④	⑤	⑥	⑦
Improve the application of the economic resources obtained toward the destination	①	②	③	④	⑤	⑥	⑦
To increase economic and financial independence in order to achieve better compliance with social objectives	①	②	③	④	⑤	⑥	⑦
To increase the financial resources obtained	①	②	③	④	⑤	⑥	⑦
Minimize the total costs of each service or activity	①	②	③	④	⑤	⑥	⑦
To increase the obtaining of requested subsidies	①	②	③	④	⑤	⑥	⑦

### Social Activity Perspective

**Table:**

Comply with the activity objectives set	①	②	③	④	⑤	⑥	⑦
Respond to the demand as soon as possible	①	②	③	④	⑤	⑥	⑦
Increase the activity carried out	①	②	③	④	⑤	⑥	⑦
Improve and maintain the specific equipment needed to meet the social objective	①	②	③	④	⑤	⑥	⑦
To promote society’s interest in the entity	①	②	③	④	⑤	⑥	⑦
Increase the efficiency of the staff	①	②	③	④	⑤	⑥	⑦

### Training and Development Perspective

**Table:**

Promoting employee training	①	②	③	④	⑤	⑥	⑦
Increase employee satisfaction	①	②	③	④	⑤	⑥	⑦
Assess the impact of training	①	②	③	④	⑤	⑥	⑦
Encourage participation	①	②	③	④	⑤	⑥	⑦
Reduce employee absenteeism	①	②	③	④	⑤	⑥	⑦
Promote the improvement of internal communication	①	②	③	④	⑤	⑥	⑦
